# Characteristics of Inclusive Web-Based Leisure Activities for Children With Disabilities: Qualitative Descriptive Study

**DOI:** 10.2196/38236

**Published:** 2023-05-04

**Authors:** Mehrnoosh Movahed, Ishana Rue, Paul Yejong Yoo, Tamara Sogomonian, Annette Majnemer, Keiko Shikako

**Affiliations:** 1 School of Physical and Occupational Therapy, McGill University Montreal, QC Canada

**Keywords:** children with disabilities, social inclusion, participation, accessibility, leisure, web-based activities, pandemic

## Abstract

**Background:**

The participation of children with disabilities in leisure activities is a key determinant of their physical and mental health. The COVID-19 pandemic has limited participation in leisure activities for all children, particularly those with disabilities. As a result, children with disabilities may be less active while feeling more isolated and stressed. Web-based communities and activities have become increasingly important. Understanding how web-based activities include or exclude children with disabilities can contribute to the development of inclusive communities that may support participation after the pandemic.

**Objective:**

This study aimed to identify factors that may facilitate or prevent the participation of children with disabilities in web-based leisure activities.

**Methods:**

We adopted a qualitative descriptive interpretative methodology and conducted interviews with 2 groups of participants: service providers offering inclusive web-based leisure activities and parents of children with disabilities who have engaged in web-based leisure activities during the COVID-19 pandemic. A semistructured interview format was created based on the Theoretical Domains Framework. The questions focused on the description of the web-based activities offered by the service provider (eg, age range, frequency, cost, target population, and type of activity offered) and any adaptations to make the web-based activity accessible to children and youth with disabilities, and their perceptions and beliefs about what supported or deterred participation in the activities.

**Results:**

A total of 17 participants described their experiences in participating in and creating web-based leisure programs and the factors preventing or facilitating children’s participation in web-based activities. Environment and context factors included accommodations, the format of activities and the web-based setting, stakeholder involvement, and materials and resources available. Activities that had flexible schedules, both recorded and live options for joining, and that provided clear instructions and information were perceived as more accessible. Beliefs involved the characteristics of the child and the family environment, as well as the characteristics of the organizations providing the activity. Activity facilitators who were familiar with the web-based environment and knew the specific characteristics of the child facilitated their participation. Engagement in community champions and respect for children’s individual preferences were perceived as positive. Access to technology, funding, and caregivers’ ability to facilitate child engagement are crucial factors that must be considered when offering web-based programs.

**Conclusions:**

Web-based environments offer an accessible and safe option for leisure participation when public health conditions prevent children with disabilities from participating in in-person activities. However, to make web-based activities accessible to children with a variety of disabilities, there needs to be a clear plan toward universal web-based accessibility that accounts for individual needs and collective approaches to web-based leisure. Future work should consider developing and testing guidelines for web-based accessibility, equity, public policy, and programming considerations in offering these activities for all children.

## Introduction

### Background

Participation in leisure activities is a human right as per the United Nations Conventions on the Rights of the Children statement that “accessible and inclusive environments and facilities must be made available to children with disabilities, to enable them to enjoy their rights” (Article 31; page 5) [1]. In addition, it is indicated by the United Nations Conventions on the Rights of Persons with Disabilities to “ensure that children with disabilities have equal access with other children to participation in play, recreation and leisure and sporting activities, including those activities in the school system” [2].

Leisure refers to the time designated for freely chosen activities performed outside self-care or work (school) [3]. Participation in leisure activities is a key determinant of the physical and mental health of children with disabilities, essential in developing skills and competencies, socializing with peers, exploring personal interests, and improving their quality of life [4-7]. However, children with disabilities are at a higher risk of exclusion from several forms of participation in leisure activities. Children with disabilities and their families face challenges in engaging in different activities and accessing essential services throughout their lives [8]. A Canadian national survey demonstrated that among children with disabilities in Canada aged 0-4 years, 69.7% reported mild or moderate difficulty while playing, and 8.8% reported severe difficulty while playing. Among children aged 5-14 years with disabilities, 44.3% reported disadvantages in transportation or leisure, illustrating the many barriers that exist for children with different disabilities in engaging in leisure environments [9].

With the rapid spread of COVID-19 the World Health Organization declared the outbreak a public health emergency of international concern on January 30, 2020; and on March 11, 2020, it was declared a global pandemic [10]. Countries worldwide, including Canada, began massive health campaigns to protect the public through prevention protocols and control interventions to limit the spread of the virus. These measures include self-isolation, social distancing, and stay-at-home recommendations. Accordingly, public and private facilities were obligated to close, which also led to a sudden change in the availability of leisure activities, programs, and all other essential forms of socialization for children [5].

The challenges faced by all children during the pandemic, including social isolation and disruption of daily activities, have been exacerbated in children with disabilities and their families [11]. For instance, approximately 45% of children with disabilities did not have access to in-clinic services for mental or physical health, and approximately 40% did not benefit from telehealth services for mental or physical health during the pandemic [12]. Only a minority (30%) had access to individualized educational plans [13]. Studies during the pandemic have found that lockdown restrictions have had considerable negative effects on physical activity levels and the mental and behavioral health of children with disabilities [14]. According to Statistics Canada, 50% of parents were concerned about their children’s physical activity levels. In addition, more than 60% of parents reported concerns about a lack of opportunities for their children to socialize [15].

### Objective

Technology and web-based environments were essential for optimizing communication and socialization during the COVID-19 pandemic. Adults and children shifted to web-based environments to learn, stay connected with their peers, and maintain their physical, social, and mental health [16]. Many organizations that previously led in-person leisure activities quickly pivoted to web-based platforms to continue offering options for activities such as sports, music, arts, camps, and life skills programs [15]. Allen et al [17] found that during the pandemic, parents with young children acknowledged the importance of physical activity for their children but questioned the suitability of web-based leisure activities as an option for young children. The same issue was raised by parents of children with disabilities in web-based forums and support groups during the pandemic period. Although web-based activities present as a positive alternative for maintaining physical activity, socializing, and receiving health and education services [18], little is known about the characteristics of web-based activities that can enhance inclusion and accessibility for children with different disabilities. Therefore, the objective of this study was to identify factors that may facilitate or prevent the participation of children with disabilities in web-based leisure activities and to inform the development of future inclusive web-based environments.

## Methods

### Overview

We developed an interview guide using the Theoretical Domains Framework (TDF), which was created to identify barriers and facilitators in clinical practice [19]. The TDF identifies areas that can act as barriers or facilitators to implement a certain type of intervention. For instance, it suggests individual (eg, service provider perceives their role as responsible to implement the web-based program) and organizational (eg, the organization has a culture of innovation) factors that promote or hinder the implementation of practices or programs. In this case, we applied the TDF to understand the barriers and facilitators to participation of children with in web-based leisure activities. Examples of the interview questions and their corresponding TDF domains are listed in Table 1. Multimedia Appendix 1 provides the full interview guide.

**Table 1:** Examples of interview questions and corresponding Theoretical Domains Framework (TDF) domains.

TDF	Questions
Social or professional role and identity	*Service providers*: did you consult with an expert (eg, accessibility consultant, web-based platform consultant, occupational therapist) on how to make your web-based program accessible before introducing your programs?
Beliefs about capabilities	Question to both *service providers* and *parents*: In your opinion, how accessible is the program to individuals with different activity limitations? Parents: what were the main challenges that your child faced in the web-based activities in which your child participated?
Beliefs about consequences	*Service providers*: in an ideal world, what would make this web-based activity more accessible or fully accessible or more inclusive?
Environmental context and resources	Question to both *service providers* and *parents*: Is it free or is there a fee? Requires registration or anyone can join? Is it open to “new” members or only for people who participated before COVID?

We adopted a semistructured interview format to facilitate dialogue between the researcher and participants to collect open-ended qualitative data. The questions focused on the description of the web-based activities offered by the service provider (eg, age range, frequency, cost, target population, and type of activity offered) and any adaptations that were implemented to make the web-based activity more accessible to children and youth with disabilities. Follow-up questions were asked as needed to explore participants’ thoughts and insights on the topic of inclusive web-based activities to provide the opportunity to discuss personal experiences [20].

### Participants and Procedures

Participants were selected from organizations listed as offering web-based leisure activities to children with disabilities on the Jooay App. These activities were added to the App by the Jooay App team through extensive web-based searches during the pandemic. The Jooay App is a free mobile and web app (Jooay [21]) created in Canada in 2015 [22]. It is a resource platform on which children with disabilities and their families, clinicians, educators, and others can locate leisure opportunities that are close to where they live, accessible, suit their needs and abilities, and match their preferences.

We used a convenience, purposeful maximum representation sample to recruit two distinct groups of participants: (1) service providers offering inclusive web-based leisure activities and (2) parents of children with disabilities who participated in web-based leisure activities during the pandemic. These 2 groups were selected to identify the structural and experiential aspects of the programs, representing different types of activities (eg, sports and arts) and activities tailored to different types of disabilities (eg, physical disabilities, autism spectrum disorders, and visual impairments). Children and youth were considered for interviews, but because of limitations in the ethics review board procedures during the pandemic, they were excluded from the study.

### Recruitment of Service Providers

A recruitment email, including information about the Jooay App, the objective of the study, and the steps required for participation in the study, was sent to the service providers of inclusive web-based activities in Canada, which were listed as “Online” activities on the Jooay App. The email was followed by a phone call to the publicly available phone number if no email response was received.

### Recruitment of Parents

A recruitment flyer was posted multiple times on Jooay social media platforms including Facebook, Instagram, and Twitter. Interested participants were contacted via email to schedule an interview at their convenience (phone or videoconference on Zoom platform), in their preferred language (English or French), using accessibility options, if desired. Once the interview was scheduled, the participant received both a consent form and a demographic form to be returned before the scheduled interview. The interviews took approximately 30 to 45 minutes, and each interview was led by 1 of 3 members of the research team. The interviews were recorded and transcribed verbatim for qualitative analyses.

### Data Analysis

Interview transcripts were imported into NVivo 12 (QSR International) [23], a qualitative data management software. An interpretative descriptive analysis methodology was applied to analyze the data collected in this study to understand the factors that may facilitate or hinder participation in web-based leisure activities for children with disabilities in web-based activities. First, the research team developed an initial coding scheme, based on a sample analysis of the 2 interviews. The initial themes were identified deductively (through the questions or answers, based on the TDF) and then inductively (distancing from the questions to interpret what the participants were bringing to the discussion, generalizing and creating new themes based on the interpretation gained beyond the direct questions). Two members of the team analyzed the remaining interview transcripts to refine the codebook and complete the analysis. The research team met regularly to discuss points of disagreement in the analysis and salient content emerging from the iterative analysis. Descriptive analysis of the participants’ sociodemographic characteristics was conducted using IBM SPSS Statistics 24 [24].

### Ethics Approval

This study was approved by the Institutional Review Board of the Faculty of Medicine and Health Sciences, McGill University (A00-B67-16B). All the participants provided informed consent form before the interviews. The contact information of the team members conducting the interviews was provided on the consent form, and there were any questions or concerns. The interviewer was also mindful of participants’ concerns or discomfort. Finally, the participants’ names and affiliations were removed from the analysis to ensure confidentiality.

## Results

### Participants’ Characteristics

A total of 15 interviews were conducted with a total of 17 participants (12 service providers and 5 parents; 2 interviews had 2 individuals from the same organization responding together). The sociodemographic characteristics of the participants and their children (parents of children with disabilities) are presented in Tables 2 and 3.

**Table 2:** Sociodemographic characteristics of organization representatives.

ID	Age	Education	Position	Work status
1	30	University—postgraduate degree	Recreation therapist	Full-time
2	45	University degree	Executive director	Full-time
3	52	—^a^	Executive director	Part-time
4	31	University—postgraduate degree	Teacher; therapist	Part-time
5	43	University degree	Regional manager	Full-time
6	28	Some high school education	Program’s coordinator	Full-time
7	57	University—postgraduate degree	Executive director	Full-time
8	66	University—postgraduate degree	Executive director	Full-time
9	22	University degree	Community engagement coordinator	Full-time
10	31	University—postgraduate degree	Founder; Executive Director	Part-time
11	44	University degree	Director of Operations	Full-time
12	42	University degree	Founder; Executive Director	Full-time

^a^Missing data as the participant preferred not to answer the question.

**Table 3:** Sociodemographic characteristics of parents and their children^a^.

ID	Parent’s age	Education	Annual family income	Child’s age	Type of disabilities
13	45	University degree	Between CAD $40,000 and CAD $60,000	11	Autism spectrum disorder
14	54	High-school diploma	Between CAD $20,000 and CAD $40,000	21	Intellectual + physical + Autism spectrum disorder
15	47	University degree	—^b^	11	Intellectual
16	51	University degree	Between CAD $60,000 and CAD $80,000	19	Intellectual
17	43	Postsecondary or professional degree	Between CAD $40,000 and CAD $60,000	4	Physical

^a^CAD $1 was about US $0.77 at the time of interviews.

^b^Missing data as the participant preferred not to answer the question.

The age range of children with disabilities whose parents participated in this project was 4-21 years, with a mean age of 13 (SD 6.8) years, with 3 (60%) of them being female. The types of disabilities in children included intellectual disabilities, physical disabilities, and autism spectrum disorders. Most of the organization representatives who participated in the study had more than 10 years of experience working with children with disabilities, with only 2 having between 5 and 10.

### Programs’ Characteristics

The web-based programs provided were categorized into multiple types (art, music, sports, camps, and others). Examples of web-based leisure programs included web-based zoo and museum tours, book clubs, life skills groups, and youth leadership development programs. The activities included a vast age range and types of disabilities (Table 4). The activities described were offered 1 to 5 times a week for groups of 3-10 participants with 30-90–minute duration.

**Table 4:** Characteristics of web-based leisure programs (n=34).

Characteristics of programs		Values, n (%)
**Activity type**		
	Art	2 (6)
	Music	3 (9)
	Sport	7 (21)
	Camp	3 (9)
	Others	19 (56)
**Age range^a^**		
	Preschool (0-5 years old)	2 (6)
	School age (5-18 years old)	25 (71)
	Young adult (>18 years old)	8 (23)
**Types of disabilities accommodated^a^**		
	Intellectual	3 (7)
	Physical or motor	7 (16)
	Autism spectrum disorder	7 (16)
	Visual	0 (0)
	Hearing	0 (0)
	Behavioral or mental health	1 (2)
	Communication	3 (7)
	Chronic illnesses	4 (9)
	Others	
	Attention-deficit hyperactivity disorder	1 (2)
	Cerebral palsy	8 (19)
	Developmental coordination disorder	6 (14)
	Fetal alcohol spectrum disorder	3 (7)

^a^Some programs were provided to more than 1 age group or types of disabilities; therefore, the total number in these 2 categories were more than 34.

### Factors Influencing Participation in Web-Based Activities

The interpretative descriptive analysis yielded 7 main themes under the 2 broad TDF categories of Environmental context and Resources and Beliefs as factors associated with participation of children with disabilities in web-based leisure activities. These are discussed in the following sections. These factors were divided into several subcategories, as listed in Table 5.

**Table 5:** Factors influencing participation in web-based leisure activities.

Category and subcategory				Summary points
***Environmental context and resources (TDF^a^ domain)***				
	**Accommodations**			
		Individual	Individual activities Individual adaptations made in group activities	
		General	Strong collaboration with children and families Adequate activity duration with time for engagement	
	**Format or setting**			
		Sensory considerations	Close captioning and sign language for live and prerecorded videos	
		Accessible platforms and formats	Live stream with recorded option Structured activity sequence, flexible schedule options	
		Environment	Simple background Platform safety (logins and password required, advertising free)	
		Setup	Objects needed for the activities at hand	
		Variety of programs	Different options of programs offered to meet child’s preferences	
	**Stakeholder involvement in program development**			
		Opportunities to socialize and connect with others	Family involvement in planning Collaboration with community organizations	
	**Material resources**			
		Activities	Appropriate for the child’s age, ability, and skill level Cost of activities and materials, funding options	
		Information	Clear and provided in advance	
		Technology and equipment	Verification of equipment requirements in advance	
***Beliefs (TDF domain)***				
	Characteristics of children			Limited attention span to focus on the screen Individualized needs known to activity provider
	Characteristics of the home environments and families			Parents availability to support or participate in the activity Familiarity with web-based environments
	Characteristics of the organizations			Collaborations with other organizations Interprofessional support Staff training and retention Readiness to change or respond Staff knowledgeable about activity and about individual needs of participants

^a^TDF: Theoretical Domains Framework.

### Environmental Context and Resources

#### Accommodations

Accommodations are considered as the features that help making web-based activities inclusive. Individual accommodations were those made to address specific needs of children, as described by a service provider:

We’re looking at everybody that we’re supporting on a very individual basis. So, you know, it really depends on who we’re serving.

ID#12

The participants suggested that service providers must *establish a strong collaboration* with children and parents in advance. This included incorporating input from parents about program options and their child’s needs and creating space for parents to give feedback and request specific accessibility accommodations. Examples of how this was done include creating a registration form or making individual phone calls to collect information and interact with parents before the activity. Service providers also modified their activities based on participants’ characteristics, including allowing extra time between activities so that all children could be prepared for the next activity or providing extra guidance to help a child get a movement done right. Other accommodations included *providing options for modifications*, such as the design of a standing or sitting form for a given movement. For example, one service provider mentioned as follows:

So, if I know that we are going to be doing something that is a little bit more challenging, give some form of modification so that it is all inclusive. I will demonstrate in the way that I believe everybody could potentially have the ability to do it.

ID#5

Families also contributed accommodations to facilitate their child’s participation; for example, parents used a larger screen to help the child focus and identify the optimal duration of the activity to sustain the child’s attention. Service providers in our study described *program durations* ranging from 20 minutes to 1.5 hours. A shorter duration was often preferred for children, especially in younger age groups, because of the limited attention span on a screen. It was also noted that short breaks during activities helped maintain participation.

#### Format or Setting

Participants noted how the format and setting of the web-based environment contributed to or hindered participation. Some participants indicated that live, synchronous sessions were more engaging, whereas others mentioned the benefits of recorded programs. Recorded programs allowed families to follow the program at times that might be more convenient for them, alleviating the competing demands during the pandemic:

They had shown us the super simple songs on YouTube where they have 2 to 30 minute engaging songs and videos about counting and numbers and alphabets and colors and all that type early learning stuff that I could set (my daughter) up with one of those and I could get a few things done or answer emails or do paperwork...So, that was a good one for us for me to get a break.

ID#17

Having both synchronous and asynchronous options available seemed to be the preferred format for families:

It was a Facebook live event. But if you weren’t able to make it for that time, you could go back and watch it later, which was really a big deal for us because morning is a hard time for us to get going, so we were always able to go and watch these little videos and sort of put them in throughout our day for like a little break.

ID#17

Many parents noted that web-based programs offered more flexibility than in-person activities, avoiding commute time and other accessibility issues such as parking or facing treacherous weather conditions:

Outside the pandemic, I would sign up for some of these things again. During cold and flu season in the winter when we don’t want to drag the wheelchair outside to go to a class..., like not having to move the chair in and out or clean it off the snow when you get to the place that you’re going to it was. It’s a pretty big deal.

ID#17

However, the web-based format also presents an inherent barrier to participation, for instance, parents’ familiarity with the web-based platform—*“The technology requires a certain amount of know-how” (ID#11)*—and physical presence requirements during the activity to facilitate their children’s participation. Strategies to overcome the accessibility issues in the web-based environment included offering the families a brief training on how to use the platform before the activity and sending a preactivity survey to identify, whereas participants had the technology required for participation. It also included considering the background of the program to be simple and conducive to facilitating focus on the staff facilitating the activity with few elements that could be distracting for participants.

The characteristics of the activities provided by organizations played an important role in the participation of children with disabilities. The opportunity for the child to choose from a *variety of activities* that aligned with their preferences and interests was a key motivator for participation. Parents and organizations noted that leisure and recreation activities were the activities that children enjoyed the most. Leisure provided opportunities to stay active and allowed social connections that were critical during the COVID-19 pandemic. The *appropriateness of the activity* to the child’s age and skill level was also essential for the success of the activity. In addition, participants noted that having a regular, predictable, and consistently *structured program* facilitated children’s participation:

It’s really engaging. It goes by super-fast. She doesn’t get bored, and by the time like half an hour’s long for a 4.5-year-old on the computer, right, she’s happy to be done, but they do a hello song and a goodbye song, so the kids know what’s coming at the beginning and the end.

ID#17

#### Stakeholder Involvement in Program Development

Web-based activities became a platform for children to socially connect and build communities for children and parents alike when they connect with other families. *Family involvement* in the planning and migration of activities from the in-person to the web-based environment was fundamental to facilitating participation. The web-based format was accommodating for parents and siblings to participate with the child. This family involvement created a familiar environment for the child to feel safe, where the parent can help if and when the child is “having a hard time self-regulating or with coping strategies” (ID#12), as mentioned by a service provider.

Family involvement was encouraged by the organizations by *collecting and incorporating feedback and suggestions provided by children and families* into the activities, as illustrated by a service provider:

I say, what do you want to learn? What do you feel like doing or how do you want me to help you get stronger? So, it’s a very open dialogue.

ID#10

This information was collected before the development of the program and after a few sessions. The children’s preferences were also considered, as they often determined what activity was done that day and informed further program development in a client-centered approach to service delivery as presented by a service provider: *“We always plan our activities around what our clients want to do” (ID#5)*. In addition, *collaboration with external organizations and stakeholders* was also important in designing and creating activities or programs. For instance, organizations collaborated with recreation specialists and camp directors, whereas others partnered with para-athletes to share their experiences participating in a sport and answer participants’ questions. Indeed, collaboration with other organizations was perceived as contributing to better program and activity development and helped create a more engaging environment. This was illustrated by a service provider who mentioned the following:

We have often paired with other organizations...we’ve been doing lots of partnership and so a lot of new program ideas come from what they are hopeful to see as well for their families.

ID#12

#### Material Resources

The resources described by the study participants can be divided into *financial, information, technology, and equipment*. Financial resources were a main barrier for service providers to offer quality and accessible programs and for parents to register their children. Financial support by governmental or nongovernmental organizations was a facilitator of participation; nevertheless, free activities may have reduced families’ commitment to participate. This was illustrated by one parent:

I really appreciated the fact that they were free. It gave you the option to go on, but with free programming, like someone was obviously paying for it out of a resource fund that they had, but I find that you as a participant don’t have to pay even a token 5$ or whatever it is towards it, but since you’re not paying and it’s not out of pocket for you, your level of responsibility is very low, your accountability.

ID#15

Another concern for parents was information about appropriate activities. Families highlighted the importance of clearly identifying which demographic activity was tailored to assess the fit of the activity for their child. A parent mentioned the following:

It’s been hard to figure out and navigate new programming and things that are accessible for her.

ID# 15

Another parent indicated that:

We were really, really happy with the different things that were available to us, but we are also super connected in our community and with our recreation therapists. So, I think if parents don’t have connections, they’re not gonna realize what’s out there, so they obviously need to advertise it a little bit more too. I mean, we do a lot of things by word of mouth as well.

ID#17

Adequate technology available, including a computer and reliable internet for the use of the child, was essential to engage with the web-based programs, and acted as a barrier for participation if those resources were not available, as indicated by a service provider:

One of our regular families through the years has four kids so they would send four at a time, but they don’t have four computers so they would send two at a time for virtual programs.

ID#8

### Beliefs

Beliefs about capabilities—what one knows and how it is applied into the program, and about consequences—the understanding about what happens if certain information or knowledge is used or not, were also part of how study participants described the actions taken in the web-based programs to facilitate participation in the activities.

### Characteristics of Children

Participants expressed several challenges that were unique to their child’s condition, and how the pandemic exacerbated ongoing problems, such as finding leisure activities that are suitable for their child’s needs. For example, one parent mentioned that the following:

My daughter has cerebral palsy, she’s nonverbal, and has a seizure disorder and also a cognitive delay. So, finding activities appropriate for her is very very challenging.

ID#15

However, parents’ beliefs on the importance of leisure, and the outcomes to their child, motivated them to make the necessary efforts to have their child engaging:

“I have two daughters with special needs. It’s been challenging, but as I said, before the pandemic, she was very social, she has lots of friends, but one of the main problems, the friends that she need, she does end up connecting with them a lot online, through Facebook and other social media, because they live all over the city”

ID#16

and that despite parents’ own experience in navigating web-based activities, *“We are a bit zoomed-out” (ID#16).*

In addition, as the pandemic situation evolved rapidly, it was challenging for children to clearly understand the situation and why they had to switch to web-based activities instead of in-person settings. For children with intellectual disabilities and younger children, parents mentioned that limited attention span to focus on the screen and lack of understanding that other people on the screen were engaging in the activity with them in real time were barriers to engagement. For example, one parent mentioned the following:

The second session he started having a tantrum because he didn’t understand what was going on, why I was keeping him in front of the screen.

ID#13

Parents suggested that the activity should be very stimulating and adopt different formats to help the child stay focused. However, parents noticed an improvement over time as their child adjusted to the web-based format, demonstrating increased confidence in the program as familiarity with the format grew.

### Characteristics of the Home Environment and Families

Parents reported that their child’s experience of the activity was influenced by the *presence of parents, siblings* or other support workers with the child during the activity. Adult involvement included facilitating the use of technology, creating space for the child to perform physical activity, and communicating with the activity facilitator on behalf of the child. For activities targeted at younger children, parents felt it was helpful to have activities in which they had a specific role, similar to the therapist’s role during an in-person activity. Although *willing to engage* and believing in the value of providing these activities for their children, parents were juggling multiple roles during the pandemic; therefore, the need to be present at the child’s web-based activity was sometimes perceived as an added stressor, whereas in-person activities were seen as opportunities for respite:

Parents have to be a lot more hands-on. It is a burden on us as parents.

ID#14

Activity providers supported parents’ beliefs in their limited capabilities and created resources in response:

What we’ve done is we’ve taken videos of kids doing activities so that we can then share them so that families can imitate it ‘cause obviously parents are facilitators in these activities. They are there with the child to manage the technology but also to make sure the kid stays put. So, the way we’re presenting it is trying to engage parent and child together in the activity, just as if it would be a therapist and a child.

ID#11

### Characteristics of the Organizations

For organizations, the impact of COVID-19 was reactionary in nature. Although some web-based activities existed before the pandemic, organizations either started promoting the existing web-based activities more actively, added more features to these web-based activities, or began offering web-based activities in response to restrictions and closures. For some organizations, this shift to a web-based environment was quick, as children with multiple disabilities were considered particularly susceptible.

It was evident that organizations had different levels of readiness to switch from in-person to web-based activities during the COVID-19 lockdown. The presence of an interdisciplinary team of health professionals and educators, such as recreational, occupational, and music therapists and early child educators, facilitated a quicker transition into web-based sessions. A *multidisciplinary team* accelerated planning for activities that were most appropriate for the organization’s clientele, accommodating individual needs and sustaining participation throughout the program season. For example, a service provider indicated as follows:

Everyone in the team that we have has different expertise, and also is an artist themselves, so that was never lost. They’re always thinking like how we can still make this you know, creative and fun.

ID#4

*Organization leadership* that also resourced into volunteers and had a wider community support had positive experiences to report:

These are university students for the most part, they know some of the kids ‘cause they’ve worked with them in the previous summer and they’re just their camp counselors, so they’re creative and flexible and motivated and enthusiastic, and that’s been a real asset to the program.

ID#2

Certain organizations hired staff who were once participants in their activities themselves. Having the participants come back as camp leaders ensured that the personnel working at the organizations were very familiar with the environment. Perceived benefits were participants reporting a feeling that this was a safe environment, and people with disabilities were represented as leaders.

*Organizations readiness* to change was also reflected in their ability to navigate web-based environments and resources. Organizations that felt familiar with their clientele did not undergo formal training to make the web-based activities accessible but rather gathered tips on how to make the web-based version of their activities accessible. Other service providers received training specific to group facilitation through Zoom to tailor activities using that platform, communicate in a text-based environment, and prompt the children to interact in specific ways to ensure flow during the activity. Nevertheless, the abrupt transition to a web-based environment was challenging for all organizations. They had to mobilize resources quickly and take considerable time and effort. These challenges were overcome through a firm belief in the organization’s missions and value of the services provided to maintain children’s health and well-being:

It’s not camp at all, and we’ve been very forward and upfront with our community that this is not what we do, but we want to stay connected and build community and build those connections. It’s crucially important. It is a challenging time for mental health and depression and social isolation and anxiety. And so, if we can bring a teeny tiny little bit of the Magic of camp into their [kids] homes and also encourage them to like get outside, get active, make some new friends...People are making new friends and we’re getting some of the benefits, we are fulfilling our mission just in a very, very different way.

ID#2

*Staff knowledge of the activity* and about the child’s and families’ needs contributed to creating programs that were trusted by parents and perceived as beneficial by the organization. Service providers described strategies they believed helped maintain attention and facilitate engagement such as understanding children’s cues, knowing when to take a break, or including extra breaks in a program when there were many participants who found it challenging to stay focused. *Making information available* for families and sending related resources (eg, links to samples of the web-based activity or related videos to gauge children’s interest in the activity, instructions for the session, and lists of required materials) to parents ahead of time helped parents adjust their child’s expectations and structure the home environment to optimize participation. This was accomplished by email, posting on the organization’s website, social media, or mail. Some organizations used mobile apps, where the list of resources and calendar of events were updated regularly and categorized by the type of activity, as illustrated by one parent:

They provide a calendar of events that they are having every month and things that repeat every month. That’s something that’s really handy when you are now planning extracurricular activities,..., it’s very important to have access to what’s available.

ID#15

*Staff’s attitudes* that supported inclusion included acknowledging the presence of all participants, ensuring that everyone felt included, and ensuring a good connection with all participants. Some service providers recruited student volunteers to cofacilitate and support the lead instructor during the program. One parent mentioned the following:

So, they really just make an effort to acknowledge that he’s there and it’s a really interesting skill to be able to speak to him as though he can speak but at the same time understanding that he’s not going to speak. That’s actually a pretty good skill to have.

ID#14

In addition, *collaborations among organizations* allowed for extended access to programs for clients and enhanced the belief in staff’s capabilities by creating leadership, mentorship, interprofessional support, and knowledge sharing opportunities for staff across organizations:

We would often partner with organizations that would recruit us to help them develop more inclusive programming, implement the programming and then train their staff.

ID#12

## Discussion

### Principal Findings

The COVID-19 pandemic has increased barriers to participation in leisure activities and other essential activities for children with disabilities [14,25,26]. The lack of access to facilities and in-person activities rapidly increased the demand for the creation of web-based communities, activities, and programs to promote health and well-being during these challenging times [16,18]. This study underscored the importance of web-based activities in promoting and maintaining physical and mental health during the COVID-19 pandemic and identified key environmental and contextual factors contributing to the participation of children with diverse disabilities in web-based programs, as well as parents’ and service providers’ beliefs that contribute to child participation in these programs. These characteristics included staff training and preparedness, use of accessible technology and provision of information for participants, variety of offers of activities with a structured and familiar sequence, ability to integrate children’s individual needs into group settings, inclusion of parents and other caregivers in program development, and interprofessional and cross-organization collaborations.

This study also showed that although organizations and parents struggled with the abrupt migration from in-person to web-based environments because of the pandemic, there was also a perceived advantage of these programs. Web-based environments can contribute to increased participation by providing community building and socialization opportunities without the physical and social accessibility barriers of in-person activities. Previous studies have demonstrated the positive influence of web-based peer mentorship programs on social engagement and participation in life situations for children with disabilities [27]. However, many web-based resources designed for children are usually not accessible to children with disabilities and require close supervision by a parent or caregiver, thus limiting participation [14]. Despite the benefits and inevitable need to develop web-based activities during the pandemic, this format is not always preferred by youth who desire social connection and have functional limitations that may limit their written or verbal communication that is prioritized in web-based environments [28]. This study confirmed this through parents’ concerns over the need for adult mediation and limited socialization opportunities in web-based activities.

Staff training and the ability to respond to children’s evolving needs have been identified as crucial aspects of inclusive leisure programing [29] and were perceived as an essential component for program creation during the COVID-19 pandemic. Client-centered activities and programs are crucial for creating inclusive web-based environments. This includes considering child and family preferences in relation to leisure participation, while considering the context of a family’s values and other personal and environmental factors related to the child [25,30]. Service providers should ensure that every activity is prepared so that participants can meaningfully engage with encouragement and support through a clear description of the activity and instructions provided in advance. The duration of the activities should be adjusted to the web-based format, age group of the children, and number of children partaking. Our results suggest that the optimal duration of activities involves an interplay between the activity type, the skill level of the child, the familiarity of the moderator (staff) with the web-based environment, and the characteristics of participants, the platform used, and the presence or absence of other facilitators who are physically with the child. In line with a previous study, our participants suggested that smaller groups of participants in each program could help children engage more meaningfully in activities [31].

Access to information about programs and dissemination of information to potential users are known determinants of participation [32,33]. During the pandemic, families dealt with a large amount of evolving information about public health measures and updates. The amount of information, coupled with the disruption in daily routines, lockdowns, and reduced social support and services, were a major source of distress for all, but particularly for families of children with disabilities [13,15]. Families can benefit from clear information tailored to their needs and from trusted sources, so they do not need to rely on generic search sites for accurate information. Leisure is often perceived by families as a source of well-being for their children and respite for parents [4], but these effects may be extinguished if they cannot access the necessary information to allow for their child’s participation and face technological barriers such as inaccessible platforms or no access to the internet. A collaborative approach to equitable access to technology that forms partnerships across sectors—education, health, community serving persons with disabilities, and web-based and mobile app listings—is an essential step for service providers and governments to be aware of [34]. In a separate study, we found that listing detailed information about existing web-based leisure activities increased the search for these activities after an all-time low search for this information during the first weeks of lockdowns in Canada [22]. We can infer that families are seeking opportunities to maintain their child’s health under all circumstances, and programs are continuously adapting and providing services that match their mission, but all operate under limited resources and strenuous circumstances. Therefore, public health initiatives are needed to bridge these resources and make information available to those who need it.

Sociodemographic and equity considerations cannot be overlooked when discussing web-based programs. Participants in this study emphasized the challenges of having technology equipment available exclusively for their child when parents were often working from home and the lack of other support that would allow for participation. In a recent study in Canada, we identified a decreased number of offerings of in-person community leisure activities in neighborhoods with higher social and material deprivation [35]. Web-based activities require access to technology and the internet. Canada has recently acknowledged a “national connectivity gap” where rural Canadians face the daily challenge of slower, less reliable internet access than those in urban centers [36]. Only 37% of rural households have access to internet speeds necessary for contemporary use, whereas only 24% of households in indigenous communities have access to such speeds. Furthermore, it was found that those from lower socioeconomic backgrounds were less likely to have a computer at home [37]. Although this finding was from 2003, it raised the ongoing concern for sufficient technology or devices or both for current internet use, particularly when there are multiple users at once. In our study, families with 1 device in their home had to share and allocate time for its use for each member of the family, which means that use is limited for each member of the household, including the child who wishes to participate in web-based leisure activities.

### Limitations and Directions for Future Research

In this study, we used a small convenience sample of Jooay App users, which limits the generalizability of our findings. As is the case for qualitative studies, however, we preconize an in-depth depiction of web-based leisure experiences to gain perspectives on characteristics that can foster reflection on the context and beliefs surrounding the creation of these programs and promote greater participation of children with disabilities in leisure and the web-based world. Furthermore, as mentioned, interviews with children and youth were not feasible because of delays in the ethics review board procedures during the pandemic. Future studies should obtain the perspectives of children and youth with a variety of disabilities and age groups and in different types of programs. The information obtained in this study can guide future studies on making web-based leisure activities more inclusive for children with disabilities, developing universal accessibility guidelines for web-based activities, and, in particular, structured evaluations of the impact of universal accessibility and client-centered web-based programs on children’s participation in leisure activities. Future studies should also consider the equity and socioeconomic factors related to the growing provision of web-based services, proposing a critical reflection on who benefits from these programs and the groups that may be further marginalized through its creation.

### Conclusions

This study identified the important characteristics of inclusive web-based activities for children with disabilities and provided suggestions on how to make future web-based activities inclusive. Despite the unprecedented nature of the pandemic and the challenges faced by engaging in web-based activities, our study also revealed that there is a strong willingness to cope, adapt, and succeed in the implementation of these programs, which also testifies to the value of these activities for children with disabilities and their families. Similarly, an organization’s readiness to change and the propensity to continue offering programs they believe were essential for the vulnerable population they serve was primordial in making these programs possible.

Although our study highlighted the willingness of organizations to adapt themselves to web-based activities, their capacities, strategies, and knowledge of how to make their programs accessible to children with disabilities varied. It is conceivable that organizations offer a blend of in-person and web-based leisure activities postpandemic to maximize accessibility and flexibility. This study can inform the development of resources aimed at promoting inclusive web-based activities that can be used by both service providers and parents of disabled children in collaborative programming.

## Appendix

Multimedia Appendix 1 Interview questions.

## Abbreviations

TDF: Theoretical Domains Framework
