# Supplementary material for: Characteristics of Inclusive Web-Based Leisure Activities for Children With Disabilities: Qualitative Descriptive Study
Source: JMIR Pediatr Parent. 2023 May 4;6:e38236. doi: 10.2196/38236 (PMC10162483; doi:10.2196/38236)
Supplement: Multimedia Appendix 1 [file pediatrics_v6i1e38236_app1.doc]

**Interview questions**

Note to interviewer: Probes will only be asked as necessary. The probes (marked with an 🡪 ) are meant to stimulate discussion.

**ORGANIZATIONS, SERVICE PROVIDERS**

1. Can you please tell me a little bit about yourself and the organization you represent?
   - Prompts: number of children registered, number of children with disabilities, are the programs exclusively for children with disabilities, or to the public at large and inclusive for children with disabilities, publicly or privately funded, years in the market, variety of programs offered, disability specific, used to have how many programs prior to COVID-19, planning to maintain online activities or resume face to face activities?
2. In which city is your organization located?
3. What kind of online adapted activities are you providing?

- Arts (including music)
- Camps
- Sports
- Other (including social clubs, computer science, public spaces)
  - If more than one type, please describe each (duration, platform used, number of children/groups, etc.)

1. What is the age range of children and youth you support?
2. How accessible to different publics is the program, considering different types of activity limitations:

- Physical,
- Intellectual,
- Auditory,
- Visual,
- Behavioral,
- Autism spectrum Disorders

1. What is the frequency of your online program?
   - Is it always available (e.g., anytime, video recorded) or it is provided in a certain times per week? (e.g., scheduled live classes that are not recorded)
2. Is it free or paid? Requires registration or anyone can join?
   - Is it open for “new” members or only for people who participated before COVID?
3. In your opinion, what characteristics make your online activities accessible for children and youth with disabilities?

- The program was made to include children with (all types of) disabilities or with a particular group in mind
- The website was modified with accessibility features, e.g., large size font, accessible color contrast, audio files, pictograms, captioning, hand sign language
- There is an online instructor who is trained to provide accommodations
- Other features?

1. Did you consult with an expert (e.g., accessibility consultant, online platform consultant, occupational therapists, etc.) on how to make your online program accessible before introducing your programs?
   - if yes, what was the focus of consultation? (e.g., technical aspects, activity adaptations, materials, etc.)
2. What have been the main challenges you’ve faced to make your online program inclusive? (or to the activities in which your child participated)
   - e.g., families don’t have access to the internet, the activities require a hands on approach, children need individual supports – parent, coach, some helper to be able to access the computer/device and/or to execute the activities being taught, do not have the technical expertise to create all accessibility features (e.g. sign language interpreter), etc.
3. In an ideal world, what would make this online activity more accessible/fully accessible/more inclusive?
   - any ideas or suggestions that could help
4. Are you providing any in-centre activities as well? (During the Pandemic)
   - How accessible are these activities for children with different types of disabilities?

**PARENTS**

1. Can you please tell me a little bit about yourself and your child/children?
2. What kind of online inclusive activities have your child partake?

- Arts (including music)
- Camps
- Sports
- Other (including social clubs, computer science, public spaces)

🡪 If more than one type, please describe each (duration, platform used, number of children/groups, etc.)

1. In your opinion, how accessible to different publics is the program, considering different types of activity limitations:

- Physical,
- Intellectual,
- Auditory,
- Visual,
- Behavioral,
- Autism spectrum Disorders

1. What is the frequency of the online program?

🡪 Is it always available (e.g., anytime, video recorded) or it is provided in a certain times per week? (e.g., scheduled live classes that are not recorded)

1. Is it free or paid? Requires registration or anyone can join?

🡪 Is it open for “new” members or only for people who participated before COVID-19?

1. In your opinion, what characteristics make online activities accessible for children and youth with disabilities?

🡪The program was made to include children with (all types of) disabilities or with a particular group in mind

🡪The website was modified with accessibility features, e.g., large size font,

🡪accessible color contrast, audio files, pictograms, captioning, hand sign language

🡪There is an online instructor who is trained to provide accommodations

🡪Other features?

1. Did you consult with an expert (e.g., accessibility consultant, occupational therapists, other healthcare professionals) on how to find or become aware of online accessible program for your child/children?
2. What have been the main challenges your child faced in that online activities in which your child participated)?

🡪 e.g., families don’t have access to the internet, the activities require a hands-on approach, children need individual supports – parent, coach, some helper to be able to access the computer/device and/or to execute the activities being taught, do not have the technical expertise to create all accessibility features (e.g., sign language interpreter), etc.

1. In an ideal world, what would make this online activity more accessible/fully accessible/more inclusive?

🡪 any ideas or suggestions that could help

1. What activities you would like to have offered online (that may or may not currently exist)?
